# The Prevalence of COVID-19 Vaccine Hesitancy Among the Black Asian Ethnic Minority in New South Wales, Australia

**DOI:** 10.7759/cureus.40626

**Published:** 2023-06-19

**Authors:** Mercy Zengeni, Naomi N Briggs

**Affiliations:** 1 Medicine, Oceania University of Medicine, Brisbane, AUS; 2 Emergency Medicine, Sunshine Coast University Hospital, Sunshine Coast, AUS

**Keywords:** minority, survey, cross-sectional, vaccine hesitancy, covid-19 vaccines

## Abstract

Introduction: Vaccination provides a cost-effective approach to controlling the COVID-19 pandemic. The success of vaccination depends on global preparedness and acceptance of the new vaccines, and this is threatened by vaccine hesitancy worldwide. This study aimed to measure the prevalence of COVID-19 vaccine hesitancy, attitudes, and contributing factors in the Black Asian Ethnic Minority (BAME) of New South Wales (NSW), Australia.

Aim: This study aimed to measure the prevalence of COVID-19 vaccine hesitancy and identify contributing factors leading to vaccine hesitancy in the Black Asian and Ethnic Minority (BAME) in NSW.

Method: A cross-sectional study was conducted among the BAME community in NSW; over 12 weeks, from January 3^rd^, 2022, to March 28^th^, 2022. The study used the pre-existing previously known 5Cs model (confidence, constraints, complacency, calculation, and collective responsibility) to assess reasons for hesitancy. The questionnaire was distributed in English using social media platforms: Facebook and WhatsApp.

Results: The study received 101 respondents over 18 years from all states in Australia from BAME communities, males and females, with different educational levels, employment sectors, marital statuses, co-existing chronic medical conditions, previous COVID-19 infection status, and COVID-19 vaccine received. Of these, 56 respondents were from NSW. Our findings revealed a high prevalence of COVID-19 vaccine hesitancy among the BAME community in NSW, with 72.8% of respondents demonstrating hesitancy/reluctance due to various attitudes identified by the 5Cs model. Despite this high hesitancy, 98.2% of the participants had received at least one to three vaccine doses.

Conclusion: Even in populations with high vaccine uptake, it is still essential to address vaccine hesitancy and provide ongoing education about the importance of vaccination, particularly as new variants of COVID-19 continue to emerge and the need for booster shots may arise. This can help ensure continued protection against the virus and prevent future outbreaks.

## Introduction

The COVID-19 pandemic has caused significant disruption to the global healthcare infrastructure and other industries, leading to a costly social and economic standstill. The virus quickly spread across the globe, causing considerable morbidity and mortality. Vaccination is considered one of the top interventions in a pandemic, proven to be a tried-and-true tool to fight the COVID-19 pandemic, stop the viral spread, lower the number of people with serious side effects, and prevent deaths [1]. Vaccines enable the adaptive immune system to generate antibodies and immune memory against potential future infections without making us sick [2]. The quick distribution of COVID-19 vaccines was aimed at preventing significant health disasters.

Historically, Tuskegee's Negro syphilis research symbolized mistreatment by the medical establishment and a metaphor for deceit, misconduct if not genocide; the most disturbing feature of this experiment is the perception that it was conducted by a federal health agency, the Public Health Service (PHS; predecessor of CDC) [3,4]. Historical factors such as this medical experimentation on minority populations without informed consent and unequal access to healthcare have contributed to a legacy of mistrust and fear in minority groups.

Vaccine hesitancy implies a delay in acceptance or refusal to take the vaccine despite the vaccines being readily available, and has been named a top ten threat to global health [5,6]. Edwards et al. reported that 36% of Australians were hesitant, and 6% were resistant to being vaccinated with a safe and effective vaccine for COVID-19 if one was available [7]. Vaccine hesitancy carries negative health consequences in various communities and settings. During the past two decades, there has been a record of increased pertussis notifications, a vaccine-preventable disease in Australian adults [8]. Addressing vaccine hesitancy and promoting vaccine acceptance is crucial to preventing disease outbreaks. The current COVID-19 pandemic and public response to the COVID-19 vaccine uptake are other examples of this critical challenge [9].

Within weeks of China first notifying the World Health Organization (WHO) of the COVID-19 outbreak on December 31^st^, 2019, a rapid proclamation, explosion, and early release of viral sequences by January 7^th^, 2020, advanced work on vaccine solutions [10]. COVID-19 vaccine production breakthrough by Pfizer/BioNTech announced an efficacy of 95%, Moderna with an effectiveness of 94.5%, and AstraZeneca 70% in 2020 [11,12].

Bell et al. said participants who identified as Black, Asian, Chinese, mixed-race, or of any other race were found to be 2.7 times more likely than white participants to report refusing the COVID-19 vaccine [13]. In another study, a significant proportion of minority community adults voiced the safety and effectiveness of the COVID-19 vaccine [12]. Similarly, Robertson et al. reported that hesitancy to vaccinate was highest in the Black (71.8%) and Pakistani/Bangladesh (42.3%) and Mixed group (32.4%) compared to white British/Irish [14]. Another study showed Aboriginal and Torres Strait Islanders are more hesitant to receive COVID-19 vaccines, primarily from cultural and historical mistrust [15]. The above studies seemed consistent with each other and confirmed vaccine hesitancy of various degrees in ethnic minorities regardless of the region where the survey occurred.

**Figure 1:**
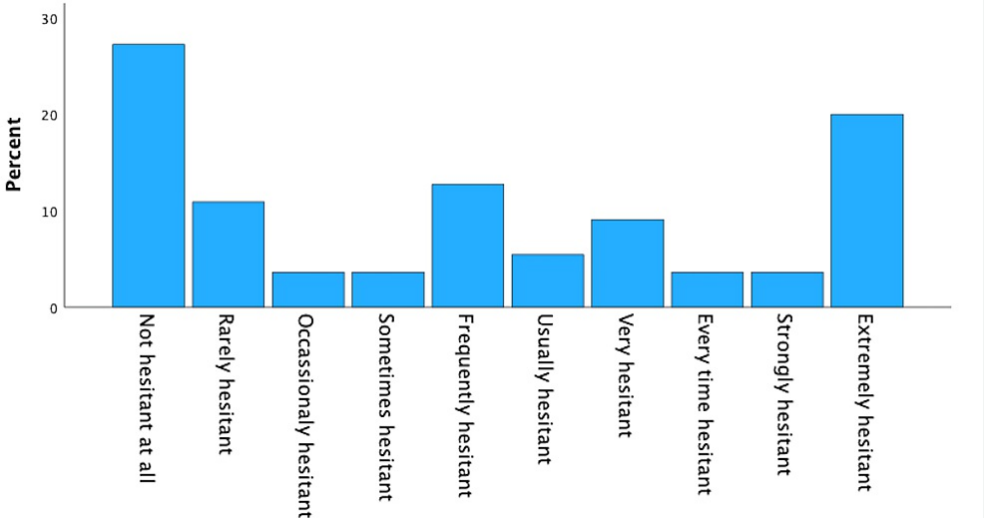
Hesitancy level

The 5Cs scale is a theoretical model that provides insight into examining psychological antecedents of vaccination, and validity relies on assumptions including collective responsibility, complacency, calculations, constraints, and confidence (Figure 1) [16]. The exact reproducibility of the processes and results to the 5Cs reliability depend on the research methodology and type of analysis, as these influence consistency and stability, especially with qualitative data [17]. Confidence primarily focuses on "Trust," which refers to the efficacy and safety of the vaccines being at the top, including reliability and competence of the delivery systems, the health services, healthcare professionals, and policymakers' motivations [18]. The "Calculation" aspect relates to an individual's involvement in comprehensive information retrieval concerning vaccination and disease risks [19,20]. Therefore, the calculation by individuals considers the risks of diseases and vaccinations [21]. The lack of knowledge or understanding can lead to distrust and fear of vaccines; however, individuals make better decisions with proper knowledge. Constraints/convenience hover around restrictions issues where "physical availability, affordability and willingness to pay, geographic accessibility, comprehension (language and health literacy), and attractiveness of immunization services affect vaccines uptake" [18]. Collective responsibility intends to protect others using herd immunity to vaccinate oneself [19]. Complacency emerges when the risk of vaccine-preventable diseases is high, and vaccination is not considered a necessary preventive measure [18].

This cross-sectional study examined COVID-19 vaccine hesitancy in the BAME community in NSW, Australia. The hypothesis was that a significant proportion of the BAME community, around 70%, may exhibit vaccine hesitancy based on similar studies from other countries [14]. Mixed methods were used in this study. The study aimed to identify the reasons for vaccine hesitancy according to the 5Cs model. Factors such as age, gender, educational level, employment status, and co-existing chronic medical conditions are some variables that were investigated as potentially being associated with vaccine hesitancy among the BAME community members.

## Materials and methods

Study location and setting

The study was cross-sectional and descriptive. A convenient sample of eligible adult participants aged > 18 years from the BAME community residing in NSW, Australia, were included in the study. Mixed methods were used for data collection.

The participants were self-selected upon seeing the research study on their WhatsApp and Facebook pages. We could have employed a random sampling method to help minimize selection bias. We could have extended the duration of study time to > three months and used multiple states in Australia to increase the sample size, as the study commenced during the lockdown; however, we had already chosen NSW. Generalizability could have been improved by making the questionnaire available in multiple languages. We chose social media due to its accessibility; it captures a large population more quickly than any other media type these days. We could have used multiple data collection methods like phone or face-to-face interviews, but no funding, and we had to observe state and nationwide lockdown restrictions.

The >18 years participants were chosen as the vaccination program was only available for this age group. The study was conducted from January 2022 to March 2022 over 12 weeks.

Ethical consideration

The study obtained approval from the International Review Board (IRB) and was conducted to meet the requirements of the Oceania University of Medicine MD program.

Anonymity was maintained throughout the study. Participants were provided with a study description, and by clicking "Continue," they offered their informed consent to participate. No personally identifiable information was collected or stored by the researchers. IRB approval number 21-1210MZ.

Materials

We collected data specifically from respondents located in all states and obtained a total of 101 responses. Among these, 56 responses were from individuals in NSW. After careful examination, we identified that one response was incomplete or had missing information, resulting in a final sample size of 55 respondents from NSW. Participants were contacted via social media, Facebook, and WhatsApp groups. The questionnaire had closed and open-ended questions, ten demographic questions, and 15 questions about COVID-19 vaccines and COVID-19 vaccine hesitancy adapted from [22] and adjusted to suit. (Appendix 1). The questionnaire included categorical, grouping, nominal, and ordinal variables for collecting quantitative data. Demographic variables had age, gender, nationality/ethnic origin, education level, and marital status, while the dependent variable was hesitancy towards COVID-19 vaccine measures on a scale of 1-10, from 1 not hesitant to 10 =extremely hesitant.

Inclusion criteria

The study included male and female adult participants aged >18 years from the BAME community residing in NSW.

Exclusion criteria

The study excluded participants below the age of 18 years and participants from states other than NSW.

Data analysis

The sample was N=55. The missing data from other states was disregarded. Descriptive statistics were used to show the responses and distribution, summarised in tables and one graph. We ran a frequency to check the closeness of variables mean, median, and mode; they were close to each other, indicating normal distribution. Numerically they were similar except for hesitancy level, but not significantly different; however, most people N=15 (27.3%) were not hesitant. Logistic regression analysis was used to identify the relationship between the dependent variable and some independent variables. Descriptive statistical frequencies of some of the variables were noted in Table 1. Qualitative data responses to questions about "reasons for vaccine hesitancy level" are presented in Figure 1. The data responses were categorized according to the 5Cs model by two independent investigators and are shown for reference, see Table 3. Statistical analysis was conducted using SPSS version 28.0. A p-value of 0.341 was observed. This indicates that the observed data did not provide strong evidence against the null hypothesis, greater than 0.05, so the results are not statistically significant.

## Results

Responses were received from 101 participants from across Australia. Data from other states was disregarded. The final sample comprised respondents from the BAME community residing in NSW (N=55) who responded to 25 questions, with one ignored due to incomplete answers. When qualitative responses for reasons for vaccine hesitancy were categorized according to the 5Cs, N=40 (72.7%) reported hesitation, and some of the respondents gave multiple reasons for reticence, with 48 hesitancy responses, refer to Table 1 for details.

**Table 1:** Descriptive statistics. Demographic and characteristics of the study R-Race-Nationality recorded

Variable	Respondents	Frequency	Percent
Age	18-29 years	6	10.9
	30-39 years	20	36.4
	40-49 years	18	32.7
	50-59 years	10	18.2
	60-69	1	1.8
Gender	Female	39	70.9
	Male	16	29.1
R-Race/nationality	Africans	45	81.8
	Asians	7	12.7
	Aboriginal Torres Strander Islander	2	1.8
	Multiracial	2	3.6
Education	Year 10/Equivalent	2	3.6
	Year 12/Equivalent	2	3.6
	Certificates/Diploma	6	10.9
	Degree	27	47.3
	Higher Education Level	19	34.5
Coexisting chronic medical problems	Yes	9	16.1
	No	45	81.8
	Unsure	1	1.8
Employment	Full time	28	50.9
	Part-time	17	30.9
	Self-employment	10	18.2
Marital status	Married	39	70.9
	Single	11	20
	Divorced	5	9.1
COVID-19 infection individual	Yes	16	29.1
	No	37	67.3
	Unsure	2	3.6
Knowledge of different COVID-19 vaccines	Yes	55	100
COVID-19 vaccination	Yes	54	98.2
	No	1	1.8
Vaccination doses	No vaccine	54	98.2
	1 dose	1	1.8
Doses received	0	1	1.8
	1	2	3.6
	2	34	33.9
	3	34	60.7
Healthcare Worker	Yes	31	56.4
	No	24	43.6
5Cs classification	Confidence	20	52.6
	Convenience/constraints	3	7.8
	Complacency	2	5.2
	Calculations	11	28.9
	Collective response	2	5.2

COVID-19 vaccine hesitancy level was reported on a scale of 1-10, with 72.7% being hesitant on the same scale (2 to 10), 27.3% having no hesitation, and 1.8% no response. Please refer to Figure 1 for further details.

When qualitative responses for reasons for vaccine hesitancy were categorized according to the 5Cs, 72.7% reported hesitation, and some of the respondents gave multiple reasons for reticence, with 48 hesitancy responses. Typical examples of participant statements according to the 5Cs about hesitancy are echoed below. Some examples relating to confidence 52.6% reported safety concerns regarding vaccine testing and thorough evaluation, the efficacy of the vaccine's effectiveness, worry about potential side effects, the rapid production of the vaccine, and conspiracy theories circulated, decreasing confidence. In calculations, 28.9% expressed a lack of knowledge about how the vaccines work or the science behind the vaccines, leading to hesitation while searching, and some were disturbed by rapidly changing information. For instance, with a complacency of 5.2%, some respondents failed to acknowledge and underestimate the importance of vaccination in controlling the spread of COVID-19. Participants expressed concerns about vaccine trial time length contributing to vaccine hesitancy.

Regarding convenience and constraints, 7.8% expressed that factors such as needle phobia and perception of low infection rates can contribute to a reduced sense of urgency for vaccination. For a collective response, 5.2% had an understanding of vaccines and vaccinations and confidence in decision-making and taking the vaccine. It's important to note that attitudes and perceptions vary widely among individuals and populations. Please refer to Table 2 in the Appendix.

Regression analysis

A logistic regression model was used to identify relationships between the dependent variable, vaccine hesitancy level, age, gender, educational level, employment status, and co-existing medical conditions. This was not significantly different from the intercept-only model, χ2 (13)= 14.475, p=.341 This indicates that the predictor variables did not significantly improve the model fit. Model fitting information is in Table 2.

**Table 2:** Model Fitting Information

Model	-2 log-likelihood	Chi-Square	Sig
Intercept only	205.197		
Final	190.722	14.475	0.341

Overall, the independent variables used in the analysis did not significantly predict the dependent variable of vaccine hesitancy. Vaccine hesitancy was independent of demographic features of age, gender, educational level, employment status, and co-existing medical conditions.

## Discussion

This study covered 12 weeks after vaccination had already commenced. All the respondents were knowledgeable about more than one type of COVID-19 vaccine in Australia, and (98.2%) had already received the COVID-19 vaccine ranging from one to three doses, which is high vaccine acceptance. Despite this, there was high vaccine hesitancy (72.7%), delaying vaccine uptake.

Despite the widespread availability of COVID-19 vaccines, a significant proportion of the BAME community remains unsure about the safety, vaccine efficacy, trusting the government, and health sector hindering global efforts to achieve herd immunity. According to the 5Cs, under "confidence" (36.4%), the most common reasons for vaccine hesitancy were safety, efficacy, side effects, the rapidly produced vaccine, and conspiracy around COVID-19 disease. The safety and effectiveness of a deemed 'rushed' COVID-19 vaccine are believed to be a conspiracy to control the population [12]. Similarly, Bell et al. reported that participants (16.7%) spoke of a lack of trust in vaccines, vaccine manufacturers, or governments, and participants (54.5%) expressed concern about the premature timing of vaccine development, limited research, unknown possible short-to-long-term side effects, and concerns about others response to the vaccine [13]. Another study reported common objections to vaccination, including refusal of vaccines, safety concerns, efficacy and manufacturing processes too fast to be safe, and skepticism about the risk of COVID-19 [23,24]. Likewise, another report on how public health campaigns and communication strategies on clear and concise messaging can promote vaccine acceptance significantly [25]. This study reports (5.5%) complacency, as some participants did not acknowledge COVID-19 devastating effects, and some voiced the need for more trials. Complacency can be a protective measure instead of admitting their lack of knowledge. To increase vaccine uptake, it is essential to continuously raise awareness about the dangers of COVID-19 and its virulence.

Educating the public about the importance of vaccination and addressing concerns and knowledge gaps can help increase vaccine uptake and reduce vaccine hesitancy. Rancher et al. reported on a survey where participants and interviewees preferred to 'wait and see' if the vaccination was safe before vaccinating themselves or their children [21]. Acknowledging and educating that the pandemic can cause devastating effects on anyone, regardless of their position or status, is essential. Accurate and transparent updated information is crucial in building trust, countering misinformation and conspiracy theories. Public health campaigns and communication strategies that focus on addressing attitudes and concerns with clear and concise messaging can also play a significant role in promoting vaccine acceptance. A study emphasized lack of transparency during policymaking and misinformation from the media could lead to substantial complacency as it fails to address mistrust [24]. The evolving nature of the pandemic and the vaccine development process should be addressed, as variants may create the need for subsequent vaccination. Chan et al. examined the impact of fake news and online misinformation on vaccination and reduced vaccination coverage as individuals became susceptible due to a lack of scientific education and understanding [26]. Reports on conflicting information from multiple stakeholders (healthcare systems, pharmaceutical companies, local, state, and federal health authorities) contribute to barriers and heighten vaccine hesitancy [27,28].

Effective pandemic management requires strategic planning, which helps overcome limitations by factoring in convenience/constraints. Additionally, providing access to vaccines in convenient and accessible locations can help increase uptake among those who may face barriers to accessing healthcare. Australia made it easy to access the COVID-19 vaccines leading to mass vaccination, centers/home-based care, walk-in clinics, mobile vaccination teams, pop-up clinics, and opportunistic vaccines in the emergency department and outpatient clinics [29]. Herd immunity benefits a portion of the population who must acquire immunity through natural infection or vaccination [30]. In this study, the collective response was only 5.2%, pushing toward achieving herd immunity and protecting vulnerable people who cannot be vaccinated. Targeted efforts to address knowledge gaps, educate and inform individuals and communities, considering their unique perspectives and concerns. The internet presents the potential for the public to make informed and uninformed decisions about vaccination [29].

Our recommendations include tailored communication efforts by the government to customize strategies for communities, considering their cultural and language preferences. Accessibility and understandability by providing easily understandable targeted information about vaccine safety and effectiveness are essential to close the knowledge gaps. Efforts to build trust by addressing concerns, providing transparent information, and involving trusted community leaders, healthcare professionals, and organizations in communication. It is crucial to recognize that change starts with listening to and understanding the concerns and attitudes of communities and then tailoring interventions accordingly.

Vaccine hesitancy was not explained by variables in the model, age, gender, employment status, education, and level of co-existing chronic medical infections. This cross-sectional study examined vaccine hesitancy attitudes in a minority group in a developed country. Our findings shed some light on COVID-19 vaccination hesitancy among the BAME community of NSW, Australia. Even in populations with solid vaccine uptake, it emphasizes the need for focused efforts to alleviate hesitation and encourage vaccine adoption, especially in minority communities.

Limitations

The study had potential limitations of convenience sampling and a smaller sample size. Using social media platforms may also introduce bias as the sample may not represent the entire BAME community in NSW. A short survey period and availability of the survey in English only may also limit the generalizability of the findings. A smaller sample size in regression analysis can reduce statistical power, increase standard errors, lead to overfitting, and modify the model's generalizability. We did not take any specific measures to address the limitations to enhance the generalizability due to lack of funding and COVID-19 lockdown restrictions uncertainty. We recommend repeating the study using a larger sample to confirm the current results and provide more robust evidence; use multiple sampling methods.

## Conclusions

Vaccine hesitancy remains a significant hindrance to vaccine uptake worldwide, especially within the BAME communities. The general distrust of safety, efficacy, lack of knowledge, and misinformation hinders vaccination success and rapid vaccine roll-out achievement. The 5Cs model helps identify gaps and tailor education to improve decision-making in addressing vaccine hesitancy. Surveys can be a valuable tool in identifying these gaps and providing insight into public attitudes and beliefs about vaccination, allowing public health officials to tailor their messaging and interventions to address specific concerns and misconceptions. In addition, providing accurate and reliable information can help improve vaccine acceptance and build trust in the healthcare system based on some of the findings.

## Appendices

**Table 3:** 5Cs data categorisation

Confidence	The new drug and no previous evidence that it will work.
	Because at first, when I started vaccinating, I was hesitant because I didn’t have trust, which made me think twice.
	I was not sure of the side effects.
	I wanted to know more about vaccine side effects.
	Not convinced that the vaccine is the only way to treat the disease.
	Was not sure of the side effects of the vaccine, and secondly, there were a lot of conspiracy theories going around about the origin of COVID-19.
	I wasn’t sure if I was going to have any side effects.
	I don’t believe there was enough research done.
	How quickly were the vaccines available?
	It’s a trial, these vaccines were developed within a short space of time. No. Mostly they need close to 10 years.
	Not confident.
	The possibility of developing health problems associated with the vaccine e.g. blood clotting conspiracy theories on population control using the vaccine.
	I was don't sure of the vaccine risks.
	My hesitancy was from a lack of confidence in the Australian Government, which appeared to take a lackadaisical approach to the vaccine.
	I was not sure of the side effects and had to call my parents in healthcare to confirm that there was no issue in getting all the available doses of the vaccine.
	Getting sick after the vaccine.
	New vac not enough research.
	I don’t believe there was enough research done to guarantee the integrity of the vaccine.
	It seems there is not enough information out there, and we are still a bit worried about how quickly the vaccine came out; most of us know people that have had terrible reactions to the vaccines.
	Concerns regarding side effects.
	Not sure of the side effects on human beings.
	Not sure of the side effects.
	Insufficient information about vaccine research and trials.
Convenience/constraints	Don't understand all the news.
	Not related to the vaccine but related to the needle.
Complacency	I also did not think at the time it was necessary to be vaccinated because the infection rates were very low at that time.
	Not necessarily. Just caution, wanted media scrutiny 1^st,^ then await a few million or so trials.
	Myth.
	The efficacy of the covid vaccine to prevent infection, why do I need to get a vaccine that’s not going to prevent infection?
Calculations	I was not sure how my body would react to the vaccine, and reading about nurses not wanting to get it makes you think twice.
	No data about side effects is available.
	Insufficient information about breastfeeding concerns regarding side effects.
	I trusted my general practitioner, but it did not happen fast, and that created slight hesitancy.
	Not enough data.
	No research, change of DNA and gene transcription and so on.
	There was contradicting information on the vaccine concerning its efficacy and side effects.
	Little to no knowledge of the vaccines and what it really is.
	That it was developed too quickly without sufficient trials, also that it alters our DNA long term.
	It seems there is not enough information out there, and we are still a bit worried about how quickly the vaccine came out.
	Most of us know people that have had terrible reactions to the vaccines.
	Not enough research on vaccines.
	Just not sure if the vaccine was going to be effective.
	Researching for side effects.
	Not enough plain English information is given through the media.
	It’s trials, these vaccines were developed within a short space of time. Mostly they need close to 10 years.”
Collective response	Compulsory nature of work.
	I believe vaccines work to curb the disease, so I had no hesitation at all.
	As a healthcare worker in a pathology laboratory, I understand that my duty to limit the rate of transmission as much as possible, where I can this includes the use of vaccination.

**Table 4:** Research Questionnaire

The prevalence of COVID-19 Vaccine hesitancy among the Black Asian ethnic minority in New South Wales, Australia
1. Are you a resident of NSW? CircleY/N . If yes, proceed.
2. Age? Tick, 18-29 years, 30-39 years, 40-49 years, 50-59 years, 60 years and above.
3. Gender? Tick, Male, Female, Other, Prefer not to say.
4. Nationality? African, Indian, Asian, Hispanic, Pacific Islander, Hispanic, Multiracial.
5. Education? Tick, Year 10/Equivalent, Year 12/Equivalent, Certificate, Diploma, Degree, Master's Degree, PhD, Others.
6. Are you a current student? Y/N
7. Employment? Tick, Full-time, Part-time, Self-employed, Unemployed, Unemployed/stay at home.
8. Healthcare Worker? Y/N.
9. Residential area? Tick, Urban, Suburban, Rural.
10. Marital status? Tick, Married, Single, Divorced.
11. Coexisting chronic medical problems? (Free text).
12. Did you get infected with COVID-19? Y/ N, I don't know.
13. Has a member of your family or friend been diagnosed or suffered a COVID-19 infection? Y/N .
14. Do you know that there are many types of COVID-19 vaccines? Y/N.
15. Have you received COVID-19? Y/ N.
16. How hesitant were you before you had the vaccine? (Scale 1-10), choose a number.
17. Are you prepared to be laid off from work for the well-being of others if you do not take the COVID-19 vaccine? Free text.
18. Are you planning on receiving the COVID-19 vaccine by 6/12? Y/ N.
19. Are you prepared to lose the freedom to travel to other countries? Y /N.
20. Are you concerned about the healthcare system? Y/N.
21. How hesitant are you about getting a COVID-19 vaccine? (Scale 1-10) 1, not hesitant at all -10, extremely hesitant
22. What are the reasons for the hesitancy you gave? Y/N.
24. Do you feel like the media contributed to your hesitancy? Y/N.
25. If yes, how is the media contributing to your hesitancy? (Free text).
